# Corticosteroid treatment in COVID-19 patients receiving extracorporeal membrane oxygenation: benefit from rational use

**DOI:** 10.1186/s13613-024-01397-w

**Published:** 2024-10-26

**Authors:** Guangting Zeng, Zanling Zhang

**Affiliations:** 1 Department of Pharmacy, The First People’s Hospital of Chenzhou, Xiangnan University, Chenzhou, China; 2 Department of Pharmacy, Xiangya Hospital, Central South University, Changsha, China

In a recent issue of Ann Intensive Care, we read with great interest the article by Ling et al. [1] who conduct a study to investigated the association between immunomodulators and mortality for patients receiving extracorporeal membrane oxygenation (ECMO) for COVID-19. Their research revealed that immunomodulators, in particular corticosteroids, were associated with signifcantly higher mortality amongst patients receiving ECMO for COVID-19. Due to the clear association between inflammatory dysregulation and adverse clinical outcomes, international guidelines unanimously recommend supporting the use of corticosteroids in critically ill COVID-19 patients. This study by Ling et al. seems to raise the alarm about whether glucocorticoid therapy should be used in COVID-19 patients receiving ECMO. Is it true that COVID-19 patients receiving ECMO do not benefit from glucocorticoid therapy? Here, we highlight some points of the study that deserve attention.

First, information on the number of enrolled patients at each site was not provided, but more than 500 sites participated, with a total of 7181 patients.Thus, center imbalances may have developed, particularly in the subgroups that did not receive an immunomodulatory agent or that received another immunomodulatory agent, with an average enrollment of less than 1 patient per site per year. Since case fatality rates vary by hospital, this imbalance may affect the results.In addition, several factors known to increase mortality, including age and comorbidities, were considered. For patients receiving ECMO, patient severity, such as sequential organ failure score, was not reported but correlated with outcome. Therefore, the possibility of baseline heterogeneity between groups cannot be excluded.

Second, the study included patients between January 1, 2020, and December 31, 2021.We have to admit that due to the lack of evidence-based guidelines in the early stage of the epidemic, there are some unreasonable points in the use of glucocorticoids in patients with COVID-19, such as dose, course of treatment, timing of use, and population selection.The standard treatment recommended by current guidelines is dexamethasone 6 mg once daily for 7–10 days, starting 7 days after the onset of symptoms. Especially for severe COVID-19 patients with high inflammation, the benefits are obvious. In some cohort studies involving the population in the early stage of the epidemic, unreasonable use of glucocorticoids, such as high dose and long course of treatment, could be observed [2].Patients with COVID-19 do not benefit from glucocorticoid therapy, but suffer from it. As high doses and long courses of glucocorticoid therapy inhibit viral clearance, and may lead to high blood sugar, increasing the risk of secondary infections, such as invasive aspergillus [3]. The lack of information on steroid dosage, duration, and timing makes it difficult to properly assess the effects of steroids in this study.Furthermore, we note that the subgroup of the population receiving immunomodulators in this study appeared to have lower levels of inflammation. Because immunomodulators were administered before or during ECMO, it is unclear whether the lower level of inflammation was due to immunomodulators or to baseline conditions.Patients with low levels of inflammation at baseline may not only fail to benefit from the use of glucocorticoids but may even be impaired.This may be one explanation for the phenomenon observed in this study.

In conclusion, glucocorticoids are a double-edged sword. we believe that patients receiving ECMO for COVID-19 benefit or suffer from glucocorticoid therapy depending on whether it is used correctly.

## Data Availability

Our manuscript has no associated data.

## Declarations

### Completing interests

On behalf of all authors, there is no completing interest.
